# The role of presepsin in pediatric patients with oncological and hematological diseases experiencing febrile neutropenia

**DOI:** 10.1038/s41598-023-33094-2

**Published:** 2023-04-20

**Authors:** Sara Cerasi, Davide Leardini, Nunzia Lisanti, Tamara Belotti, Luca Pierantoni, Daniele Zama, Marcello Lanari, Arcangelo Prete, Riccardo Masetti

**Affiliations:** 1 Pediatric Oncology and Hematology “Lalla Seràgnoli”, IRCCS Azienda Ospedaliero-Universitaria di Bologna, Via Giuseppe Massarenti 11, 40138 Bologna, Italy; 2 Pediatric Emergency Unit, IRCCS Azienda Ospedaliero-Universitaria di Bologna, 40138 Bologna, Italy

**Keywords:** Biomarkers, Oncology

## Abstract

Febrile neutropenia (FN) represents one of the main complications of pediatric patients with oncological and hematological diseases. In these patients, it is crucial to identify bacterial infections. The aim of this study is to evaluate presepsin as an early biomarker of bacterial infections during FN. We compared patients with oncological and hematological diseases and a 2:1 age-matched healthy control group. In the FN group, we evaluated 4 biomarkers, namely, C reactive protein (CRP), procalcitonin (PCT), interleukin 6 (IL6) and presepsin at the onset of fever (T0) and 48 h after T0 (T1). In the control group, we only evaluated presepsin. We enrolled a total of 41 children with oncological and hematological diseases disease experiencing 50 FN episodes and 100 healthy patients in the control group. In patients with FN, we found that presepsin was significantly higher than in the control group (*p* < 0.001). However, in the FN group, we did not find a statistically significant difference between patients with and without bacteremia (*p* = 0.989 at T0, *p* = 0.619 at T1). Presepsin values at T1 were higher in patients experiencing an unfavorable outcome (*p* = 0.025). This study shows that presepsin increases in neutropenic patients, but it only revealed useful in predicting an unfavorable outcome 48 h from the onset of fever.

## Introduction

Febrile neutropenia (FN) represents one of the main complications of pediatric patients with oncological and hematological diseases. It occurs in about one-third of those receiving chemotherapy or hematopoietic stem cell transplantation (HSCT)^1,2^. Bacterial infections can be responsible for FN, therefore early biomarkers could be essential for a prompt antibiotic treatment^3,4^. Differential diagnosis with other causes of FN, such as invasive fungal infections, viral infections or other inflammatory processes can be challenging. Bacterial cultures can support the diagnosis but are limited by delays and a significantly low sensitivity^5,6^. Moreover, limited reliable tools are available to predict the progression of these infections^7^. An appropriate diagnosis can prevent unnecessary treatments and decrease health care-associated costs. Over the years, several biomarkers have been studied to support clinicians in the management of FN. Among these markers, C-reactive protein (CRP) is the only biomarker present in the validated risk stratification algorithms mentioned by the 2017 International Pediatric Fever and Neutropenia Guidelines^8^. Procalcitonin (PCT), interleukin 6 (IL6) and the molecule presepsin seemed to be promising in supporting the clinical decision-making in the management of FN in recent studies^9–11^. Presepsin is the N-terminal fragment of the soluble form of CD14, a co-receptor expressed on the surface of monocytes/macrophages, that identifies numerous ligands such as lipopolysaccharide (LPS). Once LPS binds to CD14, it stimulates the activation of the intracellular signaling, with the subsequent activation of the immune response, phagocytosis of the pathogen bound to the receptor, proteolysis and release of presepsin into the bloodstream^12–14^. Elevated circulating presepsin levels are considered an early indicator of immune system activation, as it increases in the bloodstream 2 h after the onset of the infection^12,14,15^. The role of presepsin has extensively been studied in sepsis in both adult and pediatric patients, revealing it to be an accurate biomarker^16–23^. In detail, presepsin has been shown to be a reliable biomarker for sepsis in children presenting to the Emergency Department with signs of infection and for early- or late-onset sepsis in newborns admitted to the neonatal intensive care unit (NICU)^16,23–25^. Presepsin has also been demonstrated to be a useful tool for the diagnosis of catheter-related bloodstream infections in hospitalized children with central venous catheter and to predict the length of ICU stay in critically ill patients^26,27^. Several studies, including meta-analysis, investigated the role of presepsin in adults presenting to the emergency room or admitted to the ICU with signs of sepsis^16,28^. However, reference values in the pediatric population are still missing and data on pediatric patients receiving chemotherapy or HSCT are limited. The aim of this work is to examine the utility of presepsin compared to CRP, PCT, and IL6 for detecting bacterial infections and for predicting a negative outcome in febrile neutropenia in patients with oncological and hematological diseases.

## Methods

### Patients and study design

This prospective cohort study was performed in the Pediatric Unit and in the Pediatric Oncology and Hematology Unit of the IRCCS Azienda Ospedaliero-Universitaria di Bologna from August 2020 to September 2022. The study population included oncological and hematological patients with FN and a control group. The FN group included 41 pediatric patients aged between 1 month and 18 years, affected by an oncological or hematological disease receiving chemotherapy or HSCT. Other inclusion criteria were signed written informed consent from a parent and/or legal guardian, absolute neutrophil count (ANC) less than 500 per mmc (or expected reduction of ANC to 500 per mmc in the next 24–48 h) and body temperature ≥ 38 °C in at least two determinations 1 h apart. Patients who did not develop a reduction of ANC to 500 per mmc in the next 24–48 h were excluded. The control group included 100 apparently healthy age-matched children performing blood tests including complete blood count (CBC) for routinary controls in a ratio of 2:1 in reference to the 50 FN episodes of the patients’ group. Additional inclusion criteria were a valid signed written informed consent from a parent and/or legal guardian and no fever in the last 7 days. Exclusion criteria were known congenital immunodeficiency, known neutropenia in the last 30 days and use of antibiotics in the 10 days before. According to the normal clinical practice of our Units, patients with FN were not on prophylactic antibiotic therapy and, within 60 min of the onset of fever (defined as mentioned above as body temperature ≥ 38 °C in at least two determinations 1 h apart), blood cultures were drawn and patients were started on a beta-lactam antibiotic, associated with an antibiotic covering Gram-negatives in unstable patients. Bacteremia wase defined as a positive blood culture from both the central venous catheter and peripheral veins. An unfavorable outcome was defined as the development of septic shock or the need for admission to the ICU.

### Sample collection

In the FN group, at the onset of fever (T0), prior to administration of antibiotics, CBC, presepsin, liver and renal function, CRP, PCT, IL6 and blood cultures were performed. The same analyses, except for blood cultures, were repeated at 48 h after T0 (T1). In the control group, a small amount of blood sample was obtained from the CBC vial using the Vacuette Vacudrop device for presepsin analysis. The concentration of plasma presepsin was measured with a rapid chemiluminescence enzyme immunoassay on the fully automated PATHFAST immunoanalyzer (Mitsubishi Chemical Europe GmbH). CRP was analyzed using a turbidimetric method, and PCT and IL6 were analyzed with a chemiluminescence immunoassay.

### Study ethics

The study protocol was approved by the Ethics Committee of the CE-AVEC of Emilia-Romagna, Italy (ref. number 308/2020/Sper/AOUBo) and conducted in accordance with the Declaration of Helsinki. Written informed consent was obtained from all participants/legal guardians.

### Statistical analysis

The main characteristics of patients were reported by descriptive statistics. Normality of the distribution was assessed using the Shapiro–Wilk test. Non-normally distributed continuous variables were reported as median and interquartile range (IQR), whereas categorical variables were reported as absolute numbers and relative percentages. Qualitative clinical variables were compared using Fisher’s exact or Pearson’s chi-square test, while quantitative variables were compared using the Mann–Whitney U test. Correlation between presepsin value and leukocytes was made using the Spearmann correlation. The accuracy of CRP, PCT, IL6 and presepsin to differentiate between patients with and without bacteremia and with and without an unfavorable outcome was analyzed using ROC curves analysis. The sensitivity was calculated as the number of patients with positive results divided by the number of patients experiencing the outcome. Specificity was calculated as the number of patients with negative results divided by the number of patients not experiencing the outcome. The positive predictive value was calculated by dividing the true-positive results by the total number of patients who have positive test results. The negative predictive value was calculated by dividing the true-negative results by the total number of people who have negative test results. Positive likelihood ratio and negative likelihood ratio was calculated as sensitivity/(1-specificity) and (1-sensitivity)/specificity, respectively. The diagnostic odds ratio was determined by dividing positive likelihood ratio and negative likelihood ratio. Statistically significant differences were considered when *p* value was < 0.05. All the analyses were performed using SPSS (IBM Corp. Released 2016. IBM SPSS Statistics for Windows, Version 24.0. Armonk, NY: IBM Corp).

## Results

### Patients’ characteristics

We enrolled a total of 41 children with oncological and hematological diseases experiencing 50 FN episodes and 100 healthy patients in the control group. In the control group, median participants’ age was 7.7 years (IQR 4.4–12.3 years) and median leukocyte count was 7390/mmc (IQR 6242.5–9162.5/mmc), with no CBC abnormalities.

The study population was comparable for age to the control group (*p* = 0.741). Twenty-nine of the patients underwent HSCT. Detailed clinical information about the FN group and detailed information about the HSCT is reported in Table 1. The median leukocyte count was 55/mmc (IQR 22.5–385). 46 of the 50 (92%) episodes of FN occurred during agranulocytosis (ANC < 200/mmc), 2 (4%) during severe neutropenia (ANC < 500/mmc) and 2 (4%) developed an ANC below 500/mmc in the following 24–48 h. The median body temperature was 38.2 °C (IQR 38–38.4 °C), with a median length of fever of 42 h (IQR 24–96 h).

**Table 1:** Characteristics of the study patients (CNS = central nervous system; ALL = acute lymphoblastic leukemia; AML = acute myeloid leukemia; JMML = juvenile myelomonocytic leukemia; MUD = matched unrelated donor; MMUD = mismatched unrelated donor).

	NF patients	Controls
Number of patients	41	100
Sex		
Males	33	63
Females	8	37
Age (years old)		
0–1	3	7
2–5	11	27
6–10	10	34
11–18	17	32
Diagnosis		
Solid tumor		
CNS tumor	5	
Bone sarcoma	4	
Other	5	
Blood cancer		
ALL	17	
AML	5	
JMML	2	
Benign blood disorder		
Beta thalassemia	3	
Number of patients undergoing HSCT	29	
Type		
Allogeneic	20	
MUD	11	
MMUD	2	
Haploidentical	5	
Sibling	2	
Autologous	9	
Conditioning		
Allogenic		
Busulfan + thiotepa + fludarabine	6	
Busulfan + Melfalan + Cy	5	
TBI + etoposide	5	
Thiotepa + treosulfan + fludarabine	1	
Busulfan + thiotepa + Cy	1	
TBI + fludarabine	1	
TBI + thiotepa + fludarabine	1	
Autologous		
Busulfan + Melfalan	5	
Thiotepa	4	

### Presepsin levels are increased in patients with FN compared to healthy controls

**Figure 1:**
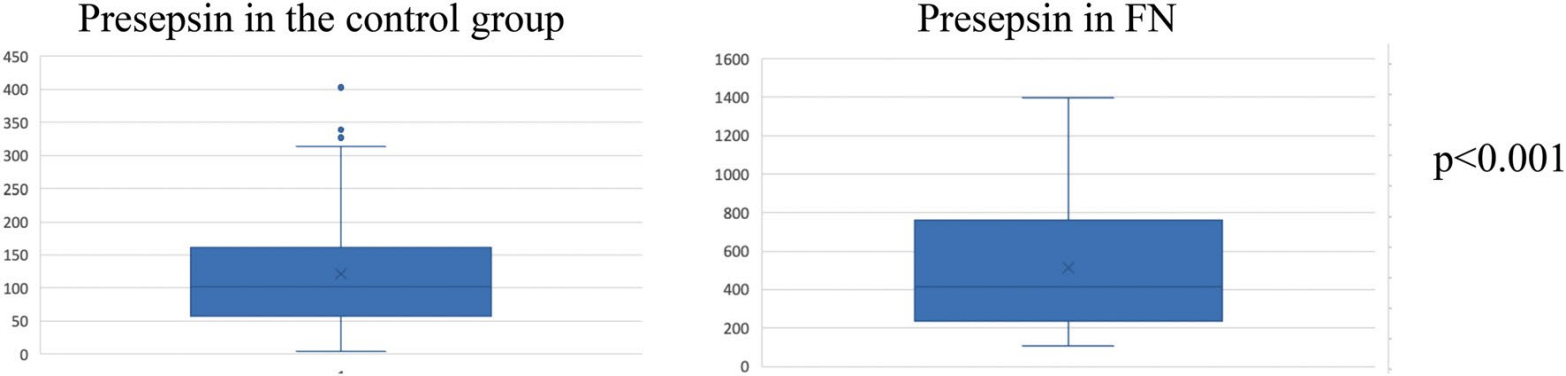
Median values of presepsin in the control group and in the FN group at T0. Figure shows presepsin values found in the control group and in the FN group.

We first analyzed presepsin values in the control group to compare these values with those of patients with oncological and hematological diseases experiencing FN. In the control group, median presepsin value was 102 pg/mL (IQR 59.2–161.3 pg/mL). In the FN group, the median presepsin value at T0 was 412.5 pg/mL, with an IQR of 250.5–761.3 pg/mL, significantly higher than the values that we found in the control group (*p* < 0.0001) (Fig. 1, Table 2). By using Spearmann correlation, we did not find a significant linear correlation between the value of presepsin and the value of leukocytes, neither in the case group at T0 or T1 nor in the control group (respectively *p* = 0.796, *p* = 0.446, *p* = 0.216) (Supplementary Fig. S1). We did not find a statistically significant difference between presepsin in patients who underwent HSCT and patients who did not (*p* = 0.989) (Supplementary Table S1).

**Table 2:** Comparison of presepsin values between controls and patients and between patients with and without bacteremia and between patients favorable and unfavorable outcome. FN: febrile neutropenia; IQR: interquartile range.

	Controls (100 patients)	FN (50 episodes)	*p* value	Bacteremia (9* episodes)	Non-bacteremia T0 (41 episodes)	*p* value	Unfavorable outcome T0 (5 episodes)	Favorable outcome T0 (45 episodes)	*p* value
Presepsin at T0 (pg/mL)									
Median	102	412.5	< 0.001	373	415	0.989	373	415	0.774
IQR	59.2–161.3	250.5–761.3		278–708	243–761.5		371–766	256.1–753	
Range	3.6–403	108–1395		191–1200	108–1395		110–1200	108–1395	
Presepsin at T1 (pg/mL)									
Median		446		359	446	0.619	933.5	398	0.025
IQR		239.2–829		152.5–1256.8	289.8–816.8		820–1837	218.3–814.5	
Range		95.5–4516		95.5–4516	181–1803		511–4516	95.5–2195	

*Among the 9 bacteremias, 2 were caused by Gram + bacteria (1 S. mitis and 1 S. aureus + M. luteus) and 7 by Gram- (3 E. Coli, 3 E. cloacae, 1 P. aeruginosa, 1 P. aeruginosa + K. pneumoniae, 1 K. pneumoniae).

### Presepsin values are not significantly higher in bacterial FN

We then analyzed the role of presepsin in distinguishing a bacterial etiology of FN. Nine of the 50 FN episodes were classified as bacteremia, defined as episodes with a positive blood culture. Detailed information about the infections is reported in Supplementary Table S2. We compared presepsin values in patients with or without bacteremia and we did not find a statistically significant difference, neither at T0 nor at T1 (respectively *p* = 0.989, *p* = 0.619) (Table 2). We also performed a subanalysis for patients undergoing or not HSCT and we did not find statistically significant differences between patients with and without bacteremia (*p* = 0.961 in the HSCT group, *p* = 0.997 in the non HSCT group) (Supplementary Table S3). Moreover, we did not find a difference in presepsin values in patients who had bacteremia from Gram-positive or from Gram-negative bacteria (*p* = 0.485 at T0, *p* = 0.206 at T1) (Supplementary Table S4).

We then calculated the ROC curves for the prediction of bacterial infections and calculated for presepsin at T0 and T1 an optimal calculated cutoff value of 410 pg/mL and 213 pg/mL, respectively. In predicting bacteremia presepsin presented a poor sensitivity (0.53) at T0 and a good sensitivity (0.90) and PPV (0.90) at T1, while it presents a poor specificity (0.55 and 0.50) at both time points (Supplementary Tables S5–S8). ROC curve analysis showed that presepsin has a low diagnostic accuracy for the prediction of blood culture positivity both at T0, with an AUC of 0.508, and at T1, with an AUC of 0.423 (Supplementary Figs. S2–S3). We compared the results with the other well-studied biomarkers and PCT is the only one who revealed to be significantly higher at both time points in the bacteremia group compared to the non-bacteremia group (*p* = 0.005 at T0 and *p* = 0.008 at T1), with an AUC of 0.793 at T0 and 0.782 at T1, representing a moderately accurate biomarker, with an optimal calculated cutoff value of 0.60 ng/mL and 3.30 ng/mL respectively (Supplementary Tables S5–S8, Supplementary Figs. S2–S3). IL6 was found to be significantly higher in the bacteremia group at T0 (*p* = 0.022) as well, being a moderately accurate test in predicting bacteremia (AUC = 0.748), but not at T1 (*p* = 0.574) (Supplementary Tables S5–S8, Supplementary Figs. S2–S3).

### Presepsin values are higher in patients with an unfavorable outcome at T1, but not at T0

**Figure 2:**
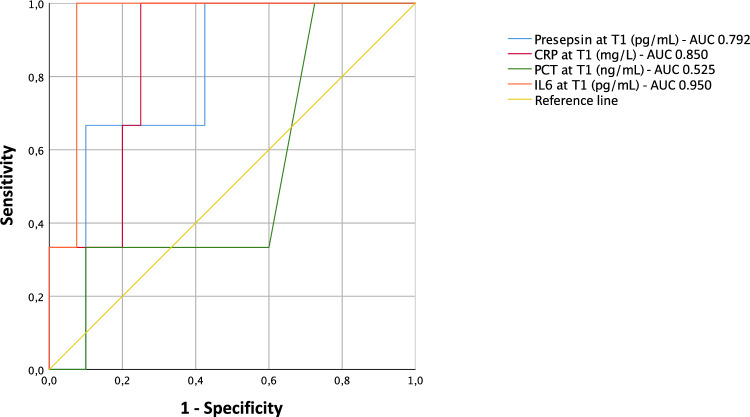
ROC curves and AUC in predicting unfavorable outcome at T1. Figure shows ROC curves of presepsin, CRP, PCT and IL6 in predicting unfavorable outcome of FN at T1.

Five of the 50 episodes of FN had an unfavorable outcome, defined as the development of septic shock or necessitating admission to the ICU. Four of these patients had bacteremia, and 2 of these patients died. We did not find at T0 a statistically significant difference between presepsin in the group with unfavorable outcome and the group with favorable outcome (*p* = 0.774) (Table 2, Supplementary Tables S9–S10). Although, with a cut-off value of 371 pg/mL, presepsin presents a good sensitivity (0.80) and a high NPV (0.95), but it has a specificity of 0.47, with an AUC of 0.546 (Supplementary Tables S9–S10, Supplementary Fig. S4). On the other side, presepsin is significantly higher at T1 in patients with an unfavorable outcome (*p* = 0.025) and, with a cut-off value of 923 pg/mL, it has a sensitivity of 0.75, a specificity of 0.89, a NPV of 0.98 (AUC 0.792) (Tables 3 and 4, Fig. 2). IL6 was the only biomarker that resulted to be significantly higher at T0 in the group with an unfavorable outcome (*p* = 0.032), representing a moderately accurate test (AUC = 0.795) and presenting a sensitivity and negative predictive value of 1 when the optimal calculated cutoff value of 130 pg/mL is used (Supplementary Tables S9–S10, Supplementary Fig. S4). At T1, IL6 has high accuracy (AUC = 0.950), with a sensitivity of 1 and specificity of 0.93 when the 439.1 pg/mL cutoff is used (Tables 3 and 4, Fig. 2). At T1 also CRP resulted significantly higher in the group with unfavorable outcome (*p* = 0.010) (Tables 3 and 4). PCT did not result significantly higher at any of the time points (Tables 3 and 4).

**Table 3:** Values of biomarkers according to the outcome of FN at T1. CRP: C reactive protein; IL6: interleukin 6; IQR: interquartile range; PCT: procalcitonin.

T1	Unfavorable outcome (n = 5)	Favorable outcome (n = 45)	*P* value
Presepsin (pg/mL)			
Median	933.5	398	0.025
IQR	820–1837	218.3–814.5	
Range	511–4516	95.5–2195	
CRP (mg/L)			
Median	28.8	10.6	0.010
IQR	18.7–38.1	4.4–16.2	
Range	16.6–38.2	0.75–31.5	
PCT (ng/mL)			
Median	3.65	0.4	0.335
IQR	0.3–49.8	0.2–1.6	
Range	0.3–178.3	0.1–36.2	
IL6 (pg/mL)			
Median	538.8	49.7	0.010
IQR	488.9–2706.9	27.3–107.3	
Range	439–4875	2.2–1039.8

**Table 4:** Sensitivity, specificity, positive predictive value (PPV), negative predictive value (NPV), positive likelihood ratio (LR +), negative likelihood ratio (LR-), diagnostic odds ratio in predicting unfavorable outcome at T1. CRP: C reactive protein; IL6: interleukin 6; PCT: procalcitonin.

	Sensitivity (95% CI)	Specificity (95% CI)	PPV	NPV	LR+	LR−	DOR
Presepsin (≥ 923 pg/mL)	0.75 (0.19–0.99)	0.89 (0.75–0.96)	0.37	0.98	6.6	0.28	23.4
CRP (≥ 16.56 mg/dL)	1 (0.39–1)	0.77 (0.61–0.88)	0.29	1	4.3	0	
PCT (≥ 7 ng/mL)	0.5 (0.07–0.93)	0.90 (0.77–0.97)	0.33	0.95	5.13	0.55	9.25
IL6 (≥ 439.1 pg/mL)	1 (0.29–1)	0.93 (0.80–0.98)	0.5	1	13.67	0

## Discussion

Presepsin levels found in patients with FN were significantly higher than the values found in the control group. The increase of presepsin levels in patients with severe leukopenia, alongside with lack of correlation between presepsin levels and circulation leukocyte count, suggests that presepsin may be produced by other cell types^29–31^. Among the cells that are thought to contribute to the production of presepsin, hepatocytes seem the most plausible hypothesis, together with resident monocytes and tissue macrophages^32–34^. While Baraka et al. demonstrated that presepsin is significantly higher in patients with bacteremia and Moustafa et al. demonstrated it to be higher in patients with infection, we, like Urbonas et al., Ozdemier et al., Arikan et al., did not find a statistically significant difference^30,31,35–40^. Agnello et al. found presepsin values to be higher in the bacteremia group only at T1, and Olad et al. found higher values of presepsin in patients with bacteremia who did not have a clinically detectable source of infection^34,38^. As in other in vivo studies, but differently from in vitro studies, we did not find a difference between presepsin levels in patients with a Gram-negative or a Gram-positive bacteremia, suggesting that it is equally released in presence of LPS and in presence of other ligands typical of Gram-positive bacteria^31,34,35,39,41^. Presepsin did not reveal to be a useful biomarker in the prediction of bacteremia, neither at T0 nor at T1, showing only at T1 a good sensitivity (0.90) and PPV (0.90) when a 213 pg/mL cut-off is used. Indeed, it revealed to be the least accurate of the analyzed biomarkers in predicting bacteremia at both time points. PCT revealed to be the most accurate at both time points in predicting bacteremia, with a sensitivity of 0.78 and 0.72 and an AUC of 0.793 and 0.782, respectively at T0 and T1. Despite CRP being the only biomarker present in FN guidelines, its values do not help discriminate between the bacteremia group and the group without bacteremia. IL6, on the other side, might be a useful biomarker in that, being higher in the bacteremia group at T0, with a sensitivity identical to that of PCT.

As in other studies, but differently from Olad et al., who found presepsin to be higher in patients who died in the following 15 days, we found at T1 a statistically significant difference between presepsin in the group with unfavorable outcome and in the group with favorable outcome^30,34,35,39^. In detail, it is significantly higher in patients with an unfavorable outcome and, with a cut-off value of 923 pg/mL, it has a sensitivity of 0.75, a specificity of 0.89 and a NPV of 0.98, representing a biomarker with a moderate diagnostic accuracy (AUC 0.792) in discriminating patients who will have an unfavorable outcome. On the other side, no differences were found at T0, but even at this timepoint it presents, with a cut-off value of 371 pg/mL, a NPV of 0.95. Given its elevated NPV, presepsin could be used to identify those patients who are at minor risk of an unfavorable outcome and to de-escalate in these patients the antibiotic therapy.

IL6 is the only biomarker that was higher at T0 in the group with unfavorable outcome, thus it may be evaluated in patients with FN, having a sensitivity and negative predictive of 1 when a 130 pg/mL cutoff is used. At T1, it maintains a sensitivity and negative predictive value of 1 and presents a higher specificity compared to T0 when a 439.1 pg/mL cutoff is used. Surprisingly, PCT does not present a statistically significant difference between the two groups at none of the time points, while CRP is a moderately accurate biomarker at T1 (AUC 0.850), with a sensitivity and NPV of 1 when a 16.56 mg/dL cutoff is used.

None of the biomarkers we studied revealed accurate in predicting both bacteremia and unfavorable outcome alone, but the use of a combination of them should be considered, also given their different kinetics. Indeed, IL6 and presepsin are the first ones to increase in peripheral blood after the onset of the infection, with a time of increasing of 1 and 2 h respectively and a peak at 4 and 3 h respectively, while PCT starts increasing at 3–4 h and peaks at 8–24 h and CRP starts rising at 4–6 h with a peak at 36–48 hours^35^. Moreover, depending the sensitivity, specificity, PPV and NPV on the cut-off chosen, more studies on larger populations are needed to find the most accurate cut-off at the different time points in children with FN. The main limitation of our study was the relatively small sample size and in particular the small number of patients with an unfavorable outcome.

## Conclusion

In our study, we were able to show that presepsin increases in neutropenic patients. Presepsin did not reveal to be useful in predicting bacteremia and it revealed accurate in predicting an unfavorable outcome only 48 h after the onset of fever, being PCT the most accurate biomarker in predicting bacteremia and IL6 the most accurate in predicting an unfavorable outcome. In conclusion, the perfect biomarker has not been found yet, but the combination of the different biomarkers we have at disposition could be useful. Further studies are needed to better understand the role of presepsin, validate normal values in the pediatric population and assess its biological function.

## Supplementary Information

Supplementary Information.

## Data Availability

The datasets used and analyzed during the current study available from the corresponding author on reasonable request.
