# Author Correction: The role of presepsin in pediatric patients with oncological and hematological diseases experiencing febrile neutropenia

**DOI:** 10.1038/s41598-023-37445-x

**Published:** 2023-06-26

**Authors:** Sara Cerasi, Davide Leardini, Nunzia Lisanti, Tamara Belotti, Luca Pierantoni, Daniele Zama, Marcello Lanari, Arcangelo Prete, Riccardo Masetti

**Affiliations:** 1 Pediatric Oncology and Hematology “Lalla Seràgnoli”, IRCCS Azienda Ospedaliero-Universitaria di Bologna, Via Giuseppe Massarenti 11, 40138 Bologna, Italy; 2 Pediatric Emergency Unit, IRCCS Azienda Ospedaliero-Universitaria di Bologna, 40138 Bologna, Italy

Correction to: *Scientific Reports* https://doi.org/10.1038/s41598-023-33094-2, published online 20 April 2023

The original version of this Article contained errors in the names of authors Sara Cerasi, Davide Leardini, Nunzia Lisanti, Tamara Belotti, Luca Pierantoni, Daniele Zama, Marcello Lanari, Arcangelo Prete and Riccardo Masetti, which were incorrectly given as Cerasi Sara, Leardini Davide, Lisanti Nunzia, Belotti Tamara, Pierantoni Luca, Zama Daniele, Lanari Marcello, Prete Arcangelo and Masetti Riccardo.

The original Article and accompanying Supplementary Information file have been corrected.

