# Supplementary material for: The role of presepsin in pediatric patients with oncological and hematological diseases experiencing febrile neutropenia
Source: Sci Rep. 2023 Apr 20;13:6464. doi: 10.1038/s41598-023-33094-2 (PMC10119114; doi:10.1038/s41598-023-33094-2)
Supplement: Supplementary file 1 — Supplementary Information. [file 41598_2023_33094_MOESM1_ESM.docx]

**SUPPLEMENTARY MATERIAL**

**The role of presepsin in pediatric patients with oncological and hematological diseases experiencing febrile neutropenia**

Sara Cerasi^1^, Davide Leardini*^1^, Nunzia Lisanti^1^, Tamara Belotti^1^, Luca Pierantoni^2^, Daniele Zama^2^, Marcello Lanari^2^, Arcangelo Prete^1^, Riccardo Masetti^1^

^1^ Pediatric Oncology and Hematology “Lalla Seràgnoli”, IRCCS Azienda Ospedaliero-Universitaria di Bologna, 40138 Bologna, Italy

^2^ Pediatric Emergency Unit, IRCCS Azienda Ospedaliero-Universitaria di Bologna, 40138 Bologna, Italy

Correspondence:

Davide Leardini; Pediatric Oncology and Hematology “Lalla Seràgnoli”, IRCCS Azienda Ospedaliero-Universitaria di Bologna, Via Giuseppe Massarenti 11, 40138, Bologna, Italy; Tel: +39 051 214 4665; email: [davide.leardini3@studio.unibo.it](mailto:davide.leardini3@studio.unibo.it).

**Leukocytes (/mmc)**

**Presepsin (pg/mL)**

**Leukocytes (/mmc)**

|  | P value | Spearman Rho |  |
| --- | --- | --- | --- |
| **Presepsin T0 – leukocytes T0** | 0.796 | -.038 | 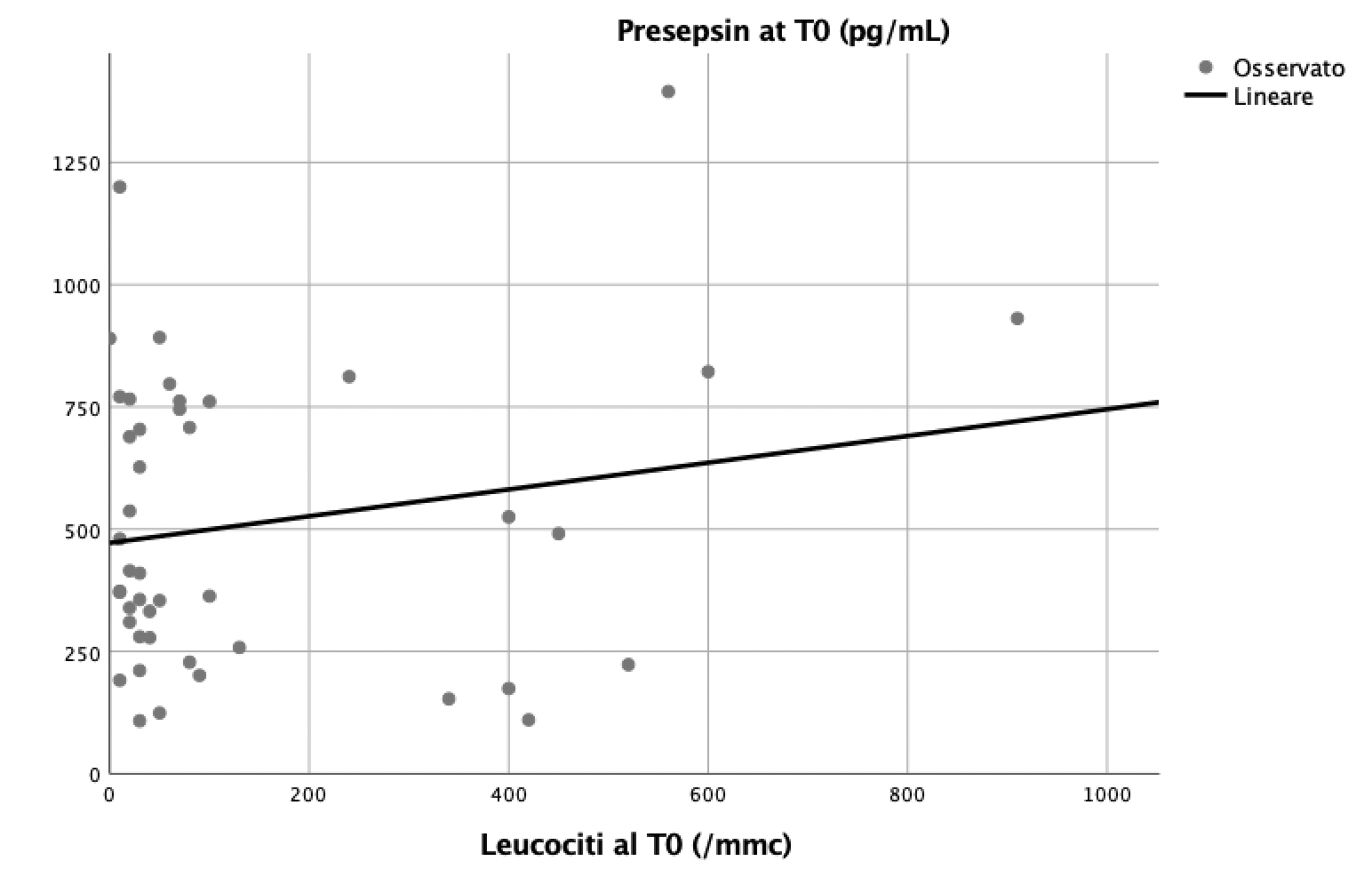  **Leukocytes (/mmc)**  **Presepsin (pg/mL)** |
| **Presepsin T1 – leukocytes T1** | 0.446 | -.114 | 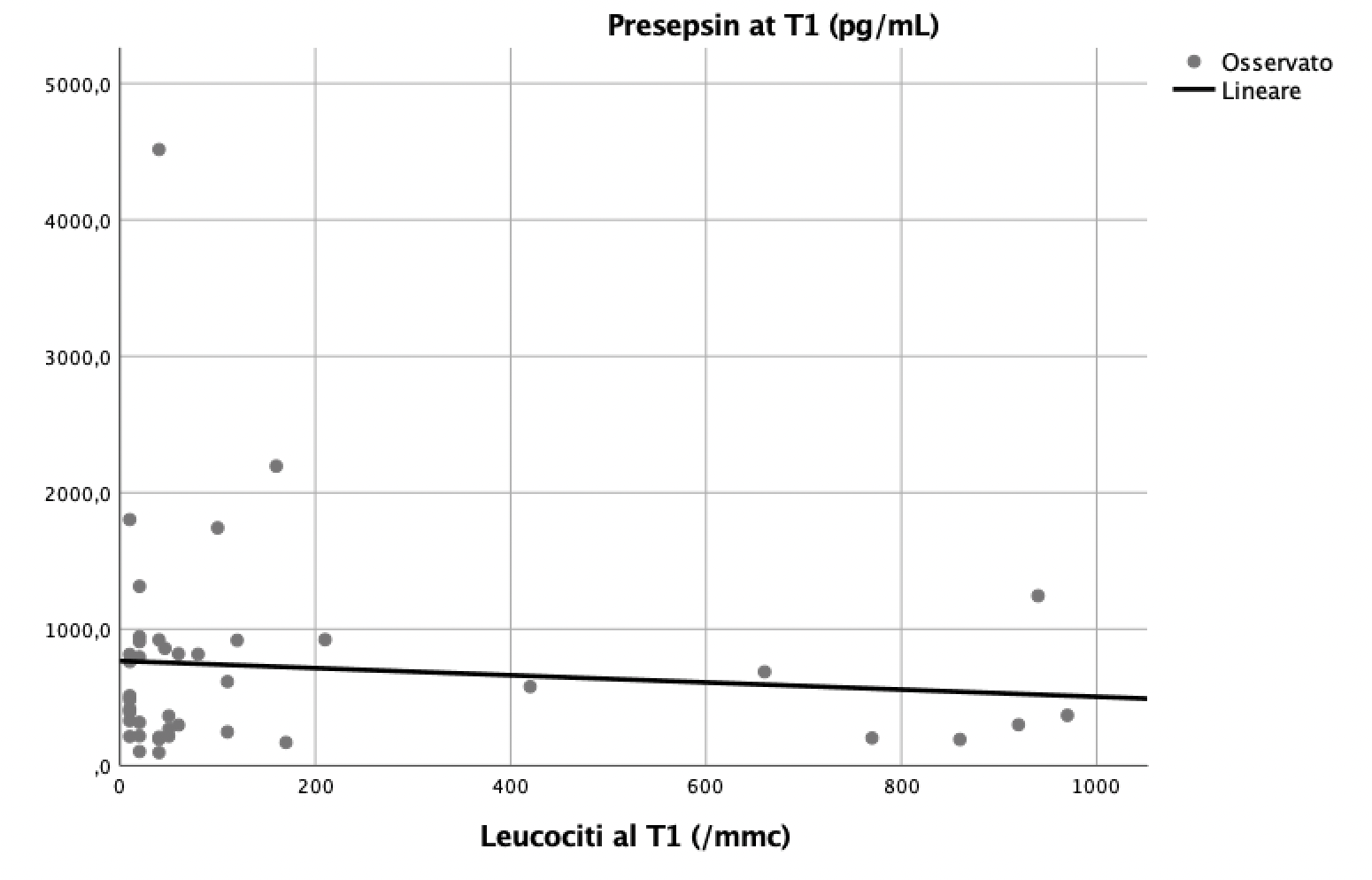  **Leukocytes (/mmc)**  **Presepsin (pg/mL)** |
| **Presepsin in controls – leukocytes in controls** | 0.216 | -.125 | 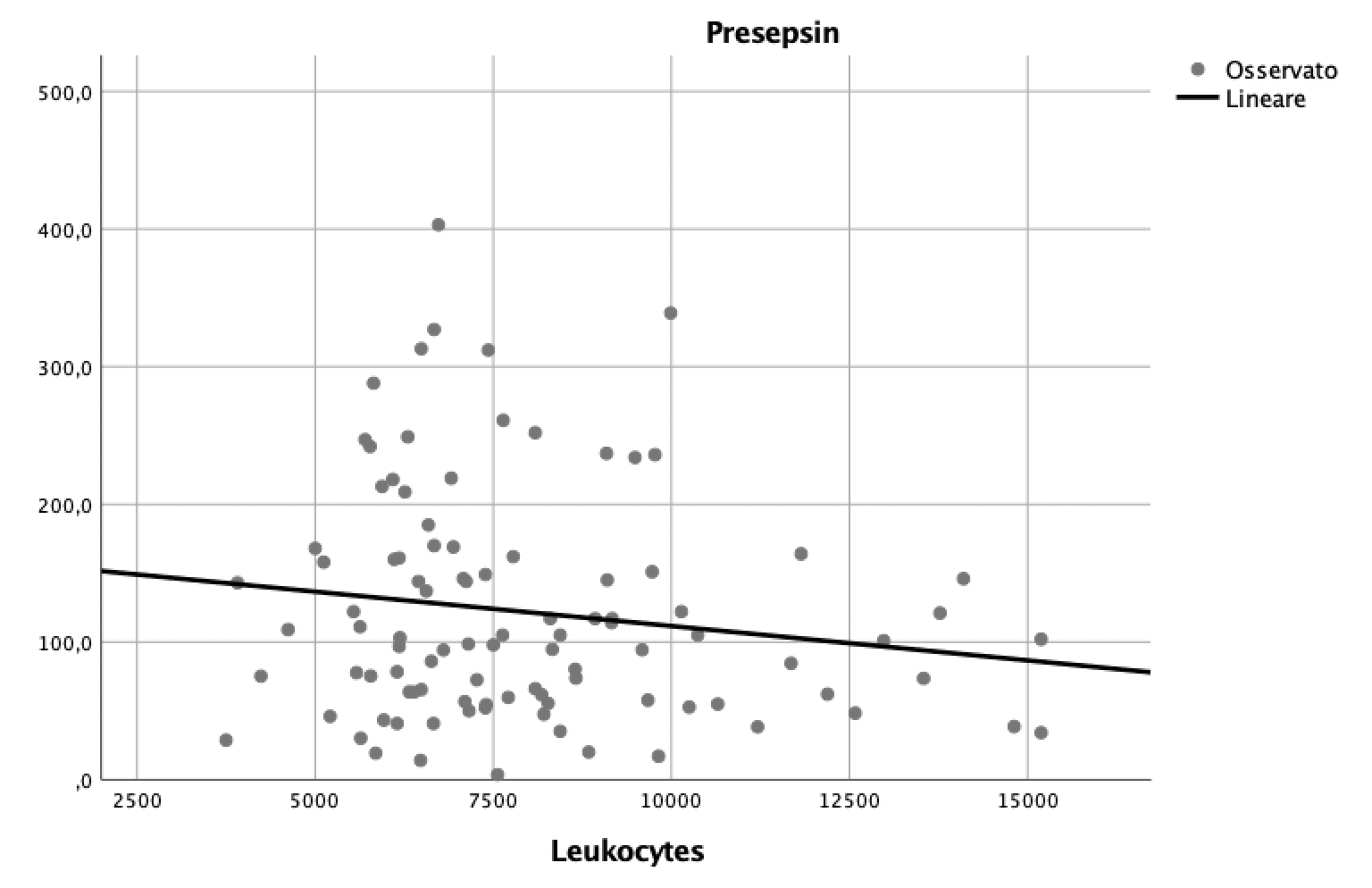  **Leukocytes (/mmc)**  **Presepsin (pg/mL)** |

**Supplementary Figure S1**: Spearmann correlation between presepsin levels and leukocytes.

**1 - Specificity**

**Sensitivity**

Reference line

- AUC 0.508

- AUC 0.610

- AUC 0.793

- AUC 0.748

**Supplementary Figure S2:** ROC curves and AUC in predicting bacteremia at T0.

**Sensitivity**

**1 - Specificity**

Reference line

- AUC 0.423

- AUC 0.739

- AUC 0.782

- AUC 0.658

**Supplementary Figure S3:** ROC curves and AUC in predicting bacteremia at T1.

Reference line

- AUC 0.546

- AUC 0.663

- AUC 0.539

- AUC 0.795

**Sensitivity**

**1 - Specificity**

**Supplementary Figure S4:** ROC curves and AUC in predicting unfavorable outcome at T0.


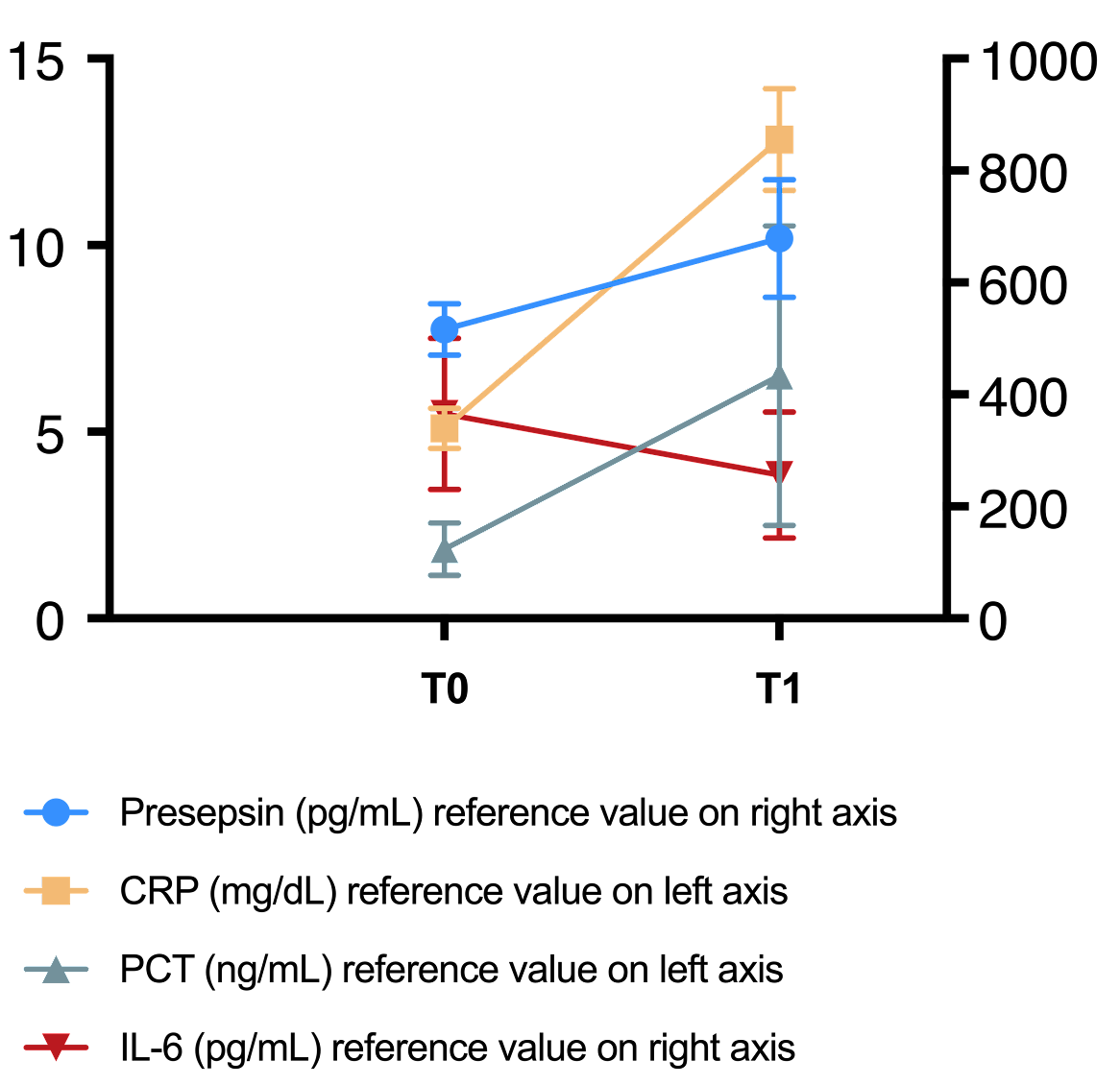


**Supplementary Figure S5:** Representation of the biomarkers’ trend.

|  | HSCT (31 episodes) | Non HSCT (19 episodes) | P value |
| --- | --- | --- | --- |
| Presepsin (pg/mL)  Median  IQR  Range | 445  315.5-765  108-3560 | 415  197.5-739.5  110-1395 | 0.989 |

**Supplementary Table S1:** Comparison of presepsin values in the subgroup analysis comparing patients who underwent HSCT and patients who did not

| Gram + | |
| --- | --- |
| Streptococcus mitis | 1 |
| Staphilococcus aureus + Micrococcus luteus | 1 |
| Gram - | |
| Escherichia coli | 3 |
| Enterobacter cloacae | 1 |
| Pseudomonas aeruginosa | 1 |
| Pseudomonas aeruginosa + Klebsiella pneumoniae | 1 |
| Klebsiella pneumoniae | 1 |

**Supplementary Table S2:** Bacterial species isolated from blood cultures

|  | Bacteremia | Non-bacteremia | P value |
| --- | --- | --- | --- |
| Presepsin in HSCT patients (pg/mL)  Median  IQR  Range | n=7  373  291-645.5  191-1200 | n=24  480  315.5-757.8  108-3560 | 0.961 |
| Presepsin in non-HSCT patients (pg/mL)  Median  IQR  Range | n=2  493  385.5-600.5  278-708 | n=17  415  194-761  110-1395 | 0.997 |

**Supplementary Table S3:** Comparison of presepsin values in the subgroup analysis comparing presepsin in the bacteremia and non bacteremia group in patients who underwent HSCT and patients who did not at T0.

|  | Gram positive bacteremia (n=2) | Gram negative bacteremia (n=7) | P value |
| --- | --- | --- | --- |
| Presepsin at T0 (pg/mL)  Median  IQR  Range | 358  274.5-441.5  191-525 | 373  324.5-737  211-1200 | 0.485 |
| Presepsin at T1 (pg/mL)  Median  IQR  Range | 99.3  97.3-101.1  95.5-103 | 944  283-1882.3  169-4516 | 0.206 |

**Supplementary Table S4:** Comparison of presepsin values in the subgroup analysis comparing presepsin in the Gram positive and Gram negative bacteremia at T0 and at T1.

| **T0** | **Bacteremia (n=9)** | **Non bacteremia (n=41)** | **P value** |
| --- | --- | --- | --- |
| Presepsin (pg/mL)  Median  IQR  Range | 373  278-708  191-1200 | 415  243-761.5  108-1395 | 0.989 |
| CRP (mg/L)  Median  IQR  Range | 5.83  2.95-10.67  1.16-12.37 | 4.25  1.65-6.36  0.3-14.03 | 0.261 |
| PCT (ng/mL)  Median  IQR  Range | 2.2  0.6-7.6  0.2-30.8 | 0.2  0.15-0.6  0.1-8.5 | 0.005 |
| IL6 (pg/mL)  Median  IQR  Range | 165.6  130.5-1172.4  77.7-4794 | 84.2  54.9-202.9  12.5-812.1 | 0.022 |

**Supplementary Table S5:** Values of biomarkers according to the cause of FN at T0.

| **T0** | **Sensitivity (95% CI)** | **Specificity (95% CI)** | **PPV** | **NPV** | **LR+** | **LR-** | **DOR** |
| --- | --- | --- | --- | --- | --- | --- | --- |
| Presepsin (≥410 pg/mL) | 0.53  (0.37-0.69) | 0.55  (0.21-0.86) | 0.84 | 0.22 | 1.21 | 0.83 | 1.45 |
| CRP (≥4.86 mg/dL) | 0.67  (0.29-0.92) | 0.56  (0.39-0.72) | 0.25 | 0.88 | 1.52 | 0.59 | 2.55 |
| PCT (≥0.60 ng/mL) | 0.78  (0.39-0.97) | 0.71  (0.55-0.85) | 0.39 | 0.93 | 2.76 | 0.31 | 8.91 |
| IL6 (≥130.50 pg/mL) | 0.78  (0.39-0.97) | 0.64  (0.47-0.79) | 0.33 | 0.93 | 2.17 | 0.35 | 6.25 |

**Supplementary Table S6:** Sensitivity. specificity. positive predictive value (PPV), negative predictive value (NPV), positive likelihood ratio (LR+), negative likelihood ratio (LR-), diagnostic odds ratio in predicting bacteremia at T0.

| **T1** | **Bacteremia (n=9)** | **Non bacteremia (n=41)** | **P value** |
| --- | --- | --- | --- |
| Presepsin (pg/mL)  Median  IQR  Range | 359  152.5-1256.8  95.5-4516 | 446  289.8-816.8  181-1803 | 0.619 |
| CRP (mg/L)  Median  IQR  Range | 17.7  11.2-27.5  1.6-38.17 | 10.56  4.4-16.5  0.75-31.48 | 0.089 |
| PCT (ng/mL)  Median  IQR  Range | 4.6  1.9-10.3  0.3-178.3 | 0.4  0.2-1.4  0.1-36.2 | 0.008 |
| IL6 (pg/mL)  Median  IQR  Range | 77  27.7-366.4  2.4-4875 | 53.1  27.6-114.4  2.2-1039.8 | 0.574 |

**Supplementary Table S7:** Values of biomarkers according to the cause of FN at T1.

| **T1** | **Sensitivity (95% CI)** | **Specificity (95% CI)** | **PPV** | **NPV** | **LR+** | **LR-** | **DOR** |
| --- | --- | --- | --- | --- | --- | --- | --- |
| Presepsin (≥213 pg/mL) | 0.90  (0.76-0.97) | 0.50  (0.16-0.84) | 0.90 | 0.50 | 1.80 | 0.20 | 9.00 |
| CRP (≥12.01 mg/dL) | 0.75  (0.35-0.97) | 0.62  (0.45-0.77) | 0.285 | 0.923 | 1.95 | 0.41 | 4.80 |
| PCT (≥3.30 ng/mL) | 0.72  (0.29-0.96) | 0.92  (0.79-0.98) | 0.63 | 0.95 | 9.05 | 0.31 | 29.17 |
| IL6 (≥293.6 pg/mL) | 0.43  (0.09-0.82) | 0.89  (0.75-0.97) | 0.43 | 0.89 | 3.96 | 0.64 | 6.19 |

**Supplementary Table S8:** Sensitivity, specificity, positive predictive value (PPV), negative predictive value (NPV), positive likelihood ratio (LR+), negative likelihood ratio (LR-), diagnostic odds ratio in predicting bacteremia at T1.

| **T0** | **Unfavorable outcome (n=5)** | **Favorable outcome (n=45)** | **P value** |
| --- | --- | --- | --- |
| Presepsin (pg/mL)  Median  IQR  Range | 373  371-766  110-1200 | 415  256.1-753  108-1395 | 0.774 |
| CRP (mg/L)  Median  IQR  Range | 7.5  4.9-10.7  1.4-12.1 | 4.25  1.65-6.36  0.3-14 | 0.201 |
| PCT (ng/mL)  Median  IQR  Range | 0.2  0.2-7.6  0.1-30.8 | 0.3  0.2-0.7  0.1-11.2 | 0.758 |
| IL6 (pg/mL)  Median  IQR  Range | 205.2  130.5-4584  130-4794 | 87.3  56-202.9  12.5-1172.4 | 0.032 |

**Supplementary Table S9:** Values of biomarkers according to the outcome of FN at T0.

| **T0** | **Sensitivity (95% CI)** | **Specificity (95% CI)** | **PPV** | **NPV** | **LR+** | **LR-** | **DOR** |
| --- | --- | --- | --- | --- | --- | --- | --- |
| Presepsin (≥371 pg/mL) | 0.80  (0.28-0.99) | 0.47  (0.31-0.62) | 0.15 | 0.95 | 1.49 | 0.43 | 3.47 |
| CRP (≥7.52 mg/dL) | 0.60  (0.16-0.95) | 0.82  (0.68-0.92) | 0.28 | 0.95 | 3.38 | 0.49 | 6.94 |
| PCT (≥7.60 ng/mL) | 0.40  (0.05-0.85) | 0.95  (0.84-0.99) | 0.50 | 0.93 | 8.6 | 0.63 | 13.67 |
| IL6 (≥130 pg/mL) | 1  (0.47-1) | 0.60  (0.44-0.75) | 0.23 | 1 | 2.53 | 0.00 |  |

**Supplementary Table S10:** Sensitivity, specificity, positive predictive value (PPV), negative predictive value (NPV), positive likelihood ratio (LR+), negative likelihood ratio (LR-), diagnostic odds ratio in predicting unfavorable outcome at T0.
